# Co-culture with normal and senescent AC16 cardiomyocytes modulates fibrotic response in primary human ventricular fibroblasts

**DOI:** 10.17912/micropub.biology.000864

**Published:** 2023-06-29

**Authors:** Veronica Hidalgo, Nikhitha Kastury, Cheyanne W. Durham, Jordan Currie, Milton Amaya, Maggie P. Y. Lam, Edward Lau

**Affiliations:** 1 Medicine, University of Colorado Anschutz Medical Campus, Aurora, Colorado, United States; 2 University of Colorado Anschutz Medical Campus, Aurora, Colorado, United States

## Abstract

Transwell co-culture with human AC16 cardiomyocyte-like cells modifies the response of primary human ventricular fibroblasts to TGF-β stimulation. Fibrotic response markers including collagen I (COL1A1) and ɑ-smooth muscle actin (ACTA2) are amplified in the presence of AC16 cells, whereas others including periostin (POSTN) and fibronectin (FN1) are suppressed. Similar modulation is observed when the ventricular fibroblasts are co-cultured with AC16 cells under baseline and induced senescence conditions. Given that the response to TGF-β stimulation is commonly measured to study fibrotic signaling and drug treatments in vitro, the results here suggest that the effect of cellular crosstalk should be more broadly considered.

**Figure 1. Fibroblast activation markers in primary human ventricular cardiac fibroblasts co-cultured with AC16 cardiac cells.**
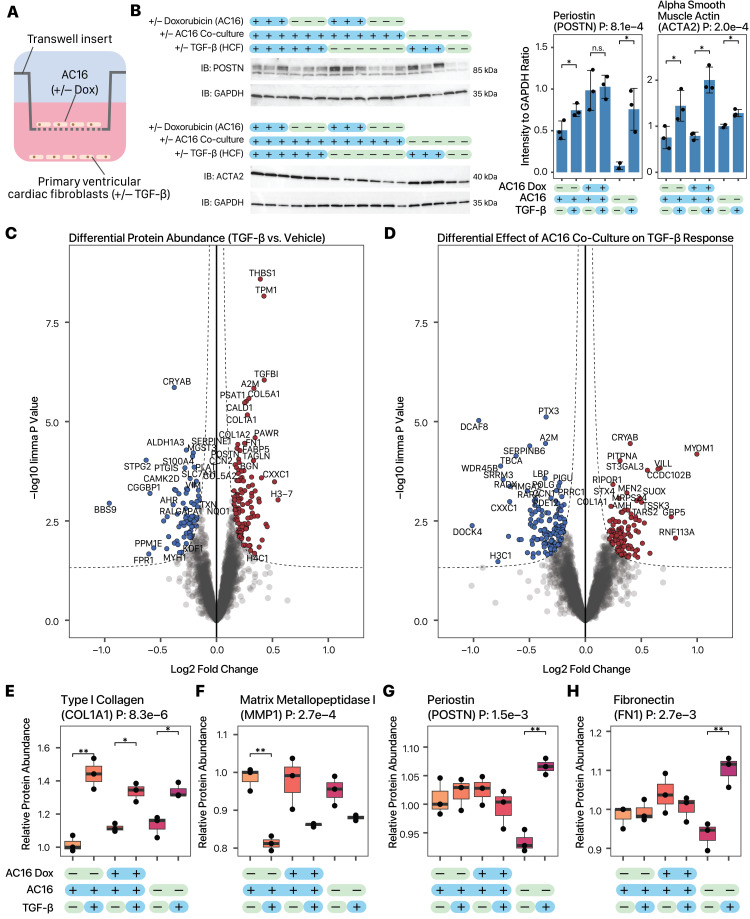
**A:** Schematic of the transwell co-culture experiment. **B:** Left: Immunoblots of fibroblast activation markers periostin (POSTN) and alpha smooth muscle actin (ACTA2). Right: Densitometry of immunoblot showing reduced periostin induction in TGF-β stimulated primary fibroblasts under co-culture with AC16 but enhanced alpha smooth muscle actin induction. n=3 for each sample except non-co-cultured TGF-β (–) fibroblasts (n=2). P values in the subtitle: ANOVA. Between sample comparison, *: t-test P value ≤ 0.05; n.s.: t-test P Value > 0.05. **C:** Volcano plots of tandem mass tag (TMT) labeling mass spectrometry experiment of TGF-β (+) vs. TGF-β (–) primary fibroblasts (n=3). X-axis: log2 fold change (doxorubicin vs. DMSO). Y-axis: –log10 of limma P value. **D:** Volcano plots of proteomics results showing the effect of AC16 co-culture modulating primary fibroblast TGF-β response (n=3). **E–H:** Relative protein abundance from mass spectrometry experiments for four fibroblast activation markers, ( **E** ) collagen type I alpha 1 chain (COL1A1), ( **F** ) matrix metallopeptidase I (MMP1), ( **G** ) periostin (POSTN), and ( **H** ) fibronectin (FN1), showing the effect of AC16 co-culture on primary fibroblast TGF-β response (n=3). P values in the subtitle: ANOVA. For between sample comparison, *: t-test P value ≤ 0.05; ** t-test P value ≤ 0.01.

## Description

Fibrosis involves the excessive deposition of the extracellular matrix and is a common feature of cardiac aging and age-associated heart diseases. The production of excessive extracellular matrix in myocardial fibrosis occurs primarily through the activation of cardiac fibroblasts. The TGF-β signaling pathway is a well characterized master regulatory signal in fibroblast activation and myofibroblast conversion (Frangogiannis, 2021) . Nevertheless, interventions that target TGF-β have not been successfully translated into anti-fibrotic therapeutics, in part due to still incomplete knowledge of the functional diversity of cardiac fibroblasts under different cellular and pathophysiological contexts. Human primary ventricular fibroblasts can be isolated and cultured in vitro for a limited number of passages, and exhibit robust responses to TGF-β stimulation in vitro, including myofibroblast marker induction and extracellular collagen deposition. To further understand the fibrotic response of human cardiac fibroblasts, we designed a co-culture experiment to determine whether the presence of human AC16 transformed cardiac cells influences the TGF-β stimulation of human primary ventricular fibroblasts. Moreover, we compared whether AC16 cells under induced senescence have a further impact on this modulation.

A co-culture experiment was designed with AC16 cardiac cells and primary cardiac fibroblasts in six different combinations of treatments: primary cardiac fibroblasts without AC16 transwell co-culture, co-cultured with normal AC16, and co-cultured with AC16 that has undergone doxorubicin-induced senescence (see companion study (Kastury et al., 2023) ) – each with or without 5 ng/mL TGF-β stimulation ( **Figure 1A** ). The transwell insert allows cellular communication through soluble factors, including secreted proteins, exosomes, and metabolites, but not direct contact of the cells. Immunoblots confirmed that TGF-β stimulation led to robust induction of alpha smooth muscle actin (ACTA2) and periostin (POSTN), two prominent fibroblast activation/myofibroblast markers. Co-culture with AC16 cardiac cells appears to have a dichotomous effect on these two markers, amplifying the activation of alpha-smooth muscle actin but blunting the induction of periostin ( **Figure 1B** ).

To acquire a more comprehensive view into the changes in fibrotic response, we performed a large unbiased proteomics screen using tandem mass tag (TMT) labeling mass spectrometry. In total, we quantified the relative abundance of 5,213 proteins in primary human cardiac fibroblasts in each of the six aforementioned conditions (n=3 each). We first assessed the proteomic profile of TGF-β stimulation in the absence of AC16 co-culture. The results showed robust differential abundance of 240 proteins at a limma P value threshold of 0.01, with 97 proteins differentially abundant at 10% FDR (42 at 5% FDR) ( **Figure 1C** ). Using a better powered linear model to dissociate the effect of TGF-β on all experimental conditions further shows 398 proteins differentially expressed at 10% FDR (229 at 5% FDR). The significantly altered proteins include known fibroblast activation markers including thrombospondin-1 (THBS1, logFC: 0.39, limma adjusted P: 1.3e–5); tropomyosin alpha-1 (TPM1, logFC: 0.42, limma adjusted P: 1.8e–5); collagen type I alpha 1 chain (COL1A1, logFC: 0.28,limma adjusted P: 3.9e–3), confirming robust fibroblast activation in this system.

We next compared the effect of AC16 on fibroblast activation, by comparing the differential abundances of proteins after TGF-β induction under co-culture conditions vs. those in baseline (without AC16 co-culture) conditions ( **Figure 1D** ). A total of 178 proteins were modulated at P values ≤ 0.01 although only 12 passed the 10% FDR cutoff. We focused on the behavior of a number of known fibrosis proteins and again observed that AC16 co-culture had a complex effect on different fibrosis markers. Both the induction of collagen type I alpha 1 chain (COL1A1) ( **Figure 1E** ) and the known repression of matrix metallopeptidase 1 (MMP1) ( **Figure 1F** ) appeared to be amplified under co-culture conditions. In contrast, the proteomics data validated the immunoblot observation of a blunting of periostin (POSTN) ( **Figure 1G** ) under co-culture conditions, and a similar trend was observed for fibronectin (FN1) ( **Figure 1H** ). Both normal and doxorubicin-treated AC16 cells, which showed P21 and thymidine kinase induction and histone loss as shown in the companion study (Kastury et al., 2023) , exerted the observed effects on the fibroblasts.

In summary, co-culture with AC16 cardiac cells has complex effects on primary fibroblast response to TGF-β stimulation, where the induction of some well-established fibroblast activation markers are boosted but others blunted. The result suggests a larger proteomic panel may be more suitable than individual markers to quantitate the degree of fibroblast activation and myofibroblast transition. An important question now is what factors from cardiac cells are responsible for this modulation. Non-fibroblasts in the heart, including cardiac myocytes, endothelial cells, and immune cells can modulate fibroblast activation via cytokines, growth factors, or extracellular matrix modulation (Bowers et al., 2022; Frangogiannis, 2021) . Because cellular communication is typically not captured in experiments that consider only single cell types in vitro, how crosstalk influences cellular processes remains little known in many physiological and pathological contexts. Notably, the data suggest that even in the absence of TGF-β, co-culture with AC16 cells also modulates periostin expression ( **Figure 1B** ). Cardiomyocytes secrete a large complement of proteins known as cardiokines that can participate in paracrine and endocrine signaling. Recent work shows a complex network of secreted proteins regulates cardiac fibrosis and TGF-β pathways (Feng et al., 2019; Ko et al., 2022; Zhang et al., 2022) . Moreover, crosstalk signals can change in aging and pathological settings (Srivastava et al., 2022; Zhang et al., 2022) . Future work will determine the secretomes of normal and senescent cardiomyocytes and identify their role in fibroblast crosstalk and TGF-β response.

## Methods

**Cell culture:**

Transformed AC16 cells were acquired from Millipore and used at P10-15 and expanded in DMEM/F-12 with 10% FBS (Gibco). Primary human ventricular cardiac fibroblasts were acquired from Promocell and expanded in FGM-3 (Promocell) and used at passage 3 – 5. For the experiment, cells were separately placed on 0.4 µm pore size transwell inserts (Greiner) at a density of 3,500 – 3,560 cells/cm ^2^ for AC16 cells and plated in normal six-well tissue-culture coated plates at a density of 3,350 – 3,550 cells/cm ^2^ for human ventricular cardiac fibroblasts. The cells were separately cultured in fibroblast growth medium FGM-3 (Promocell), which was first tested to confirm that it supported the growth of AC16 cells for at least 48 hours. Once the AC16 cells achieved 40% confluency a 0.1 µM doxorubicin- and vehicle- supplemented FGM-3 media were prepared to replace the media from four six-well plates with transwell inserts for 24 hours. Once the primary human fibroblasts achieved >10% confluence, 5 ng/mL TGF-β1 and vehicle supplemented FGM-3 media were prepared and used to replace the media from six separate 6-well-plates for 24 hours. All cells were cultured at 37°C in 5% CO _2_ . To initiate co-culture, the media in the AC16 cells was removed and the cells were washed with PBS twice; 1.5 mL FGM-3 media was added to each trans-well. The media for the six fibroblast plates was changed with fresh corresponding TGF-β1 or vehicle treated FGM-3 media for 24 hours. The trans-wells were nested into each well of the fibroblast plates according to the experimental design.

**Protein extraction and immunoblots:**

Primary cardiac ventricular fibroblasts were lysed using RIPA buffer and Protease Inhibitor (Pierce Halt) followed by sonication (Diagenode Bioruptor Pico) to solubilize proteins. Protein concentration in each sample was measured using BCA assay (Thermo) against a standard curve of bovine serum albumin. For immunoblots, a total of 30 µg extracted protein was separated on a 5–20% Mini-PROTEAN TGX precast gradient gel (Bio-Rad) and transferred onto PVDF membranes (BioRad). The membranes were blocked with 5% dried milk in TBS-T and incubated overnight with a primary antibody followed by HRP-conjugated secondary antibody as noted in the reagents table, following manufacturer protocols. Densitometry of immunoblot images were performed by one or both of two operators (V.H. and J.C.) using ImageJ v.153k (Schneider et al., 2012) and normalized to GAPDH for analysis.

**Mass spectrometry based proteomics:**

For mass spectrometry, proteins were digested using a filter-assisted sample preparation protocol. Briefly, 25 µg protein from each sample was added to pre-wetted 10 kDa MWCO 0.5 mL volume PES protein concentrator (Thermo Scientific), washed with 250 µL of 8 M urea centrifuged (10 min, 22°C, 14,000 ×g) twice to remove detergent, then exchanged to 300 µL of 100 mM triethylammonium bicarbonate (TEAB) twice followed by centrifugation (10 min, 22°C, 14,000 ×g). The proteins were reduced with 10 mM tris(2-carboxyethyl)phosphine (TCEP) and alkylated with 40 mM chloroacetamide (CAA), then digested with sequencing grade trypsin (Promega) overnight at 37 °C. Peptides were collected and labeled with TMT 10-plex reagents for 1 hr, followed by quenching with 1 µL of 5% hydroxylamine. The samples were dried with a SpeedVac concentrator, desalted with C18 clean-up columns (Thermo Scientific), then fractionated using the Pierce High pH Reversed-Phase Peptide Fractionation Kit (Thermo Scientific) following manufacturer’s protocol. The samples were analyzed on a Q-Exactive HF Orbitrap mass spectrometer coupled to an Easy-nLC 1200 liquid chromatograph (Thermo) in data-dependent acquisition mode using conventional settings as previously described (Han et al., 2022) . Mass spectrometry data was converted to mzML format using ThermoRawFileParser (Hulstaert et al., 2020) then searched using Comet v.2022_01 (Eng et al., 2015) against the UniProt SwissProt (UniProt Consortium, 2021) human protein sequence database appended with common contaminant protein sequences (retrieved 2023-03-22 using Philosopher (da Veiga Leprevost et al., 2020) ; 42,435 forward entries). Post-processing and calculation of false discovery rates (FDR) of identification was performed using Percolator (crux v.4.1 distribution) (The et al., 2016) , with 1% FDR accepted for identification. Spectrum TMT intensity was extracted using pyTMT v.0.4.1 as described (Dostal et al., 2020) .

**Statistics and data analysis**

For immunoblot data, ANOVA and unpaired two-tailed t-tests were performed to compare the means between two conditions. P value ≤ 0.05 was considered statistically significant and marked with an asterisk (*) or as indicated in the figure’s legend. Differential protein abundance analysis was performed in R v.4.2.1 using the limma package v.3.52.4 (Ritchie et al., 2015) . A limma P value of ≤ 0.01 is considered a suggestive change, whereas a FDR adjusted P of ≤ 0.1 is considered a significant result.

**Data Availability**

Raw mass spectrometry data are available on ProteomeXchange under the accession PXD041982. Uncropped immunoblot images are available on figshare at https://doi.org/10.6084/m9.figshare.22677085.

## Reagents

**Table:** 

Reagent	Manufacturer/Catalog number	Remarks
Anti-periostin rabbit monoclonal antibody	Cell Signaling #20302	1:1000 dilution
Anti-alpha smooth muscle actin rabbit monoclonal antibody (D4K9N)	Cell Signaling #19245	1:1000 dilution
Anti-GAPDH rabbit monoclonal antibody (D16H11)	Cell Signaling #5174	1:1000 dilution
Anti-rabbit IgG HRP-linked antibody	Cell Signaling #7074S	1:1000 dilution
Human TGF-β1 recombinant protein	R&D Systems 7754BH005	Used at 5 ng/mL
